# The role of ESG performance during times of COVID-19 pandemic

**DOI:** 10.1038/s41598-024-52245-7

**Published:** 2024-01-31

**Authors:** Min Gao, Xiulin Geng

**Affiliations:** School of Business, Nanjing University, No. 22 Hankou Road, Gulou District, Nanjing, 210008 Jiangsu China

**Keywords:** Environmental social sciences, Environmental sciences, Environmental impact

## Abstract

The outbreak of the epidemic in 2020 has caused a huge negative impact on the production and operation of firms, directly threatening their survival and development. However, some firms can make timely and effective adjustments in the face of sudden crises because of their resilience, and then turn the corner. This study selects the data of 2993 companies listed in Chinese A shares. The OLS method and event study is used to analyze the impact of ESG on the ability of corporate system crisis (corporate resilience). The research results indicate that companies with good ESG performance are more resilient in crises. The mechanism test indicates that the easing effect of corporate financing constraints and the expansion effect of corporate green innovation capabilities are important channels for ESG performance to promote the negative impact of crisis shocks on corporate value. Heterogeneity analysis indicates that ESG has a stronger ability to respond to systemic crises in small-scale firms, state-owned firms, and highly competitive market environments. Powerful CEOs can weaken ESG's ability to respond to systemic corporate crises. Further research has found that only S and G items, namely good governance level and social performance, have a significant positive promoting effect on corporate resilience. ESG performance may be more important in areas more severely affected by the epidemic. This study expands the research on ESG and the research on the decision mechanism of enterprise resilience. This study provides a new theoretical perspective for the study of corporate crisis response capabilities, and provides a certain policy reference for Chinese firms to effectively respond to public crises, which has important policy implications.

## Introduction

The outbreak of the COVID-19 has led to the closure of some of the governments of the world's governments in the world. This action aims to curb the spread of infectious diseases, but this may cause most companies to be in an immobilizable liquidity crisis^1^. Sudden crisis reflects the transformation, uncertainty, complexity and vagueness of the market environment has become a normal state^2^. Under this "new normal", the importance of corporate crisis response capabilities is increasingly prominent. Generating resilience in adverse events to effectively respond to crises is the key to survival and even leverage future development for firms^3^. With the outbreak of the crisis incident, the company's attention has shifted from realizing performance growth to improving crisis response capabilities, in order to resist the risks in the shock and uncertain environment to obtain survival opportunities and sustainable development^4^. How to improve the crisis response ability of an enterprise is a issue that needs to focus on the theoretical and practical circles in the post -crisis era^5^.

The systematic crisis response ability of firms is equivalent to a type of corporate resilience, which mainly includes three aspects: the ability to resist and mitigate external shocks, the ability to actively adapt to shocks and the ability to sustain development after shocks^6^. In the era of high turbulence and uncertainty, firms need to continuously reshape and expand the toughness of the enterprise, in order to effectively respond to adapting to the crisis and resume their rebound quickly, helping the enterprise to relieve difficulties, and even use this as a leverage to support the future sustainable and innovative development^7,8^.

The COVID-19 has hit many firms heavily, while some firms can turn crisis into opportunity and achieve growth against the trend due to their resilience^2^. Relevant scholars have made useful discussions on the response to the systematic crisis of the enterprise. Some studies believe that corporate governance is a key response method in the crisis period^9^. Erkens^10^ pointed out that companies with high independent directors performed even worse during the crisis. During the crisis, the concentration of ownership is positively correlated with the stock price. Compared with decentralized small shareholders, the major shareholders have more motivation to supervise management^11^. During the 1997 Asian financial crisis, companies with strong investor protection systems experienced lower stock market declines and exchange rate depreciation^12^. Essen et al.^13^ found that giving CEO compensation rewards, equity incentives, annual bonuses, etc. can enhance the firm's value during the financial crisis. Some studies have pointed out that organizational resources are important for organizational resilience^14,15^. Redundant resources can protect the technological core of an organization from the impact of environmental changes^16,17^. The cognitive factors of firms facing crises^18^, employee abilities and collective cognition^19^, corporate strategy^7^, job flexibility of organizational members^20^ and the level of digitalization of firms^21^ are factors that affect enterprise resilience. However, these studies have overlooked the advantages of enterprise ESG.

ESG not only focuses on the company's economic value, but also comprehensively considers factors such as environmental protection, social responsibility and corporate governance to promote the company's sustainable development goals and enhance the company's international image. According to estimates by the Global Sustainable Investment Alliance (GSIA), global ESG assets reached US $22.9 trillion at the beginning of 2016, accounting for 26% of the world's total assets under management, and will reach US $35.3 trillion in 2020, an increase of 54.56% from 2016 (Data comes from Global Sustainable Investment Review 2020 released by Global Sustainable Investment Alliance (GSIA).). ESG is highly consistent with China’s high-quality development goals and is an important force in promoting the sustainable development of China’s economy and achieving “carbon peak” and “carbon neutrality”.

Existing research has made useful discussions on ESG and corporate value, but scholars are controversial about the meaning of ESG. Some scholars believe that ESG practices can establish a good image and reputation for companies^5^, enhance the recognition and trust of stakeholders, ease corporate financing constraints^22^, reduce corporate risk^23^. However, some scholars regard it as an agency cost. Management may over-invest in corporate social responsibility^25^, seek personal gain at the expense of short-term interests, and abandon investment projects with positive net present values, thereby damaging the firm's value. Therefore, we aim to respond to the following research questions: how do highly rated ESG score firms perform during the COVID-19 shock? Why do they perform better than other firms in times of a pandemic shock?

The reason for the disagreement may be that research on ESG and corporate performance usually faces serious endogeneity problems, for example, there may be a reverse causal relationship between the two. Therefore, based on the above background and theoretical gaps, this study uses the exogenous shock of the COVID-19 epidemic to identify the impact of ESG on corporate systemic crisis response capabilities during the crisis and how it plays a role during the crisis, unlike previous studies based on normal environments. This study expands the research on ESG. The existing research literature on ESG mainly studies corporate innovation, corporate operating risks and corporate value based on the long-term stable external environment as implicit conditions. This study focuses on ESG's corporate resilience during crises, provides more solid data support for the conclusion that ESG improves corporate resilience, and also expands research on the determinants of corporate resilience.

The main research objectives of this study can be summarized into the following five points: (1) Relying on the existing signaling theory and agency cost theory, combined with China's special institutional background and the exogenous impact background of the COVID-19 epidemic, theoretically analyze the impact of ESG on the company's ability to respond to systemic crises, and thereby put forward reasonable research hypotheses. (2) Use OLS estimation method, fixed effects regression model, and event study method to identify the impact of ESG on corporate systemic crises, and empirically test the relationship between ESG and excess stock returns during the crisis. (3) Identify and test the mechanism and channels of ESG effects on corporates’ systemic crisis response capabilities from multiple aspects. (4) Verify whether the economic consequences of ESG performance during the crisis have brought opportunities for capital expansion to some high-quality companies and increased firm value. (5) Discuss the impact of various ESG sub-items on corporate crisis response capabilities.

The research contributions of this study are:

Explaining corporate systemic crisis response capabilities from an ESG perspective broadens the perspective and scope of corporate crisis response research. Xiao et al.^25^ studied from the perspective of corporate cash holding levels and found that there is a significant positive correlation between cash holding levels and the cumulative excess return within the event window period. The precautionary value of corporate cash holdings becomes more prominent when the external financing environment is poor. Mitton^9^ studies believe that strict corporate governance structure effectively reduces the damage to the interests of small shareholders during times of crisis. There are also scholars who mainly focus on managerial characteristics^26^ on corporate systemic crisis response. This study starts from the ESG performance issues caused by China's special institutional background and the global exogenous crisis impact, and uses a short time window to directly study ESG performance and the company's stock volatility and elasticity during crisis periods. This study provides a new perspective for companies to respond more fully to exogenous shocks.

Using the exogenous event of the COVID-19 epidemic as the background, this study provides a unique opportunity to study the impact of ESG on companies' crisis response capabilities during periods of extreme distress. It is a preliminary exploration in this field and enriches the research results of ESG-related literature. In addition, the COVID-19 crisis, as an exogenous shock event, alleviates the problems caused by endogeneity. Therefore, this study can accurately analyze the valuation effect of ESG, and to a large extent alleviate the potential endogeneity concerns of previous research, filling the theoretical gap between ESG and corporate crisis response.

This study provides practical guidance strategies for companies to consciously respond to sudden crises. With the continuous advancement of global economic integration, the international mobility of labor, capital, information and other factors is increasing. The transmission channels of the economic impact of sudden major crises are complicated, causing the social impact and economic losses caused by the epidemic to be extremely heavy. Therefore, this study is of great significance to how companies can effectively respond to crisis events and ensure corporate stability during crises.

This study provides new empirical evidence on how firms standardize governance in daily business activities before a crisis occurs. Previous research lacks literature on the differences in the functions of ESG performance under different scenarios, that is, the degree of ESG performance will be affected by environmental and other factors. This study provides a new research perspective for future in-depth analysis of corporate governance issues in the context of economic crises through the analysis of regulatory effects under different mechanisms. This study examines the inherent mechanism of ESG in corporate crisis response capabilities under extreme crises, enriching the situational conditions for ESG to play a role.

Based on this, combined with China's unique institutional background and the COVID-19 epidemic, a short time window during the epidemic was used to study the causal relationship between ESG performance and corporate crisis response capabilities. This study provides a reliable research basis for the immature crisis management field in my country.

## Literature review and hypotheses development

### Literature review

This paper connects with two main areas of research. The first area closely examines the COVID-19 pandemic's effects on financial markets^27,28^. Due to the outbreak of the COVID-19, the S&P 500 index fell by 34%, and the Hang Seng index, the FTSE MIB index of Italy and the Nikkei 225 index fell by 25%, 41% and 31% at the most^29^. The pandemic led to decreased stock values and increased market volatility^30^. Notably, Ramelli and Wagner^27^ and Albuquerque et al.^31^ observed varying stock price trends throughout the pandemic. Economic repercussions of COVID-19 extend to significant social implications, such as job disruptions, widespread unemployment, and reduced work hours in the labor market^32^.

The second strand of the literature that this paper is referred to ESG. ESG performance can enhance firm value, establish a corporate image^33^, gain competitive advantages^34^, and effectively manage and mitigate potential risks^23^. Extensive literature has focused on the daily operational activities of corporate ESG, examining its relationship with corporate performance, financing costs, and corporate risks. In terms of corporate performance, some scholars, from the perspective of stakeholder theory, argue that strengthening ESG practices helps gain the trust and support of stakeholders, thereby improving financial performance and corporate market value^35^. From the perspective of the insurance mechanism of social responsibility, companies with good ESG performance generate more moral capital, enabling them to maintain stakeholder support and consolidate trust to withstand crises, thus protecting shareholder equity^36^. Regarding financing costs, numerous studies have examined the mitigation of the cost of equity capital^37^ and debt capital^38^ through corporate ESG practices. Wong et al.^39^ investigate the impact of ESG certification on Malaysian firms, finding that ESG certification reduces a firm's cost of capital, while significantly increasing Tobin's Q. In terms of corporate risk, as ESG practices prompt companies to focus on stakeholder interests and regulate corporate behavior, they significantly reduce the risk of litigation^40^ and the probability of default, thereby alleviating credit risk^41^.

However, the studies mentioned above predominantly focus on the daily operational activities of corporations, all relating to the long-term value of these entities. There is a scarcity of literature emphasizing the short-term performance of ESG during periods of crisis. This study addresses this gap by investigating the short-term value of ESG as a signaling mechanism for companies facing crises. This research makes a significant incremental contribution by illustrating that the substantial decline in global stock values during the COVID-19 pandemic reflects intense negative sentiment among investors. This pervasive negative sentiment indiscriminately transfers in various forms. In such scenarios, ESG performance could serve as a valuable indicator, systematically mitigating negative risks during periods of crisis. This approach not only provides a nuanced understanding of ESG's role during turbulent times but also offers insights into the strategic importance of ESG practices in shaping investor perceptions and reactions in a crisis context.

### Hypotheses development

ESG plays a role by breaking the "information gap" and producing an "insurance effect". On the one hand, from the perspective of signal theory, when external stakeholders receive a signal from a company to carry out ESG practice, they can comprehensively examine the financial and non-financial information of enterprise ESG practice, break the "information gap" and reduce the perceived risk of investors^42^. By conveying positive signals, the company can improve its relationship with stakeholders and enhance investors confidence, thus affecting the market return of stocks^43^. The ESG practice of firms can be characterized as the differentiation strategy and competitive advantage of firms, which can reduce the impact of sudden exogenous events and produce 'insurance effect'^44^, and make the stock price more stable^45^. On the other hand, based on stakeholder theory and resource dependence theory, the crisis response of firms needs to draw various resources from the external environment. ESG practice can help firms obtain key strategic resources to build their own competitive advantages. When the overall trust level of society encounters an unexpected decline, companies with high social reputation and trust are more likely to receive the attention and support of stakeholders^36^, and it is easier to establish and maintain a loyal customer base, and even avoid or reduce any market value loss caused by negative events. Patten^46^ believes that corporate donations can attract investors attention and appreciation. When the market is hit by a sudden crisis, the trust from stakeholders can play an insurance role, and then the stock market will show a higher excess return rate for firms practicing ESG.

Good ESG performance can ease the financing constraints of firms. First of all, according to the signal transmission theory, firms with good performance in ESG show positive signals of sustainable development ability, which reduces the uncertainty faced by investors^47^. Secondly, corporate social responsibility is conducive to obtaining key strategic resources of stakeholders^48^, transmitting more enterprise-specific information to creditors such as banks, opening up channels for funding sources, and thus alleviating financing constraints. In addition, in the context of China's "carbon peak" and "carbon neutrality", firms with good ESG performance will receive more policy support, thereby easing financing constraints^49^. Liquidity is the key to affecting the ability of firms to cope with the crisis during the epidemic. Crisis response is a resource-consuming activity. Abundant cash is conducive to playing the role of insurance. Firms with high financing constraints will directly affect their investment activities due to cash flow constraints^50^. Therefore, ESG performance will promote the improvement of enterprise value through the alleviation of financing constraints.

Good ESG performance helps to improve the green innovation ability of firms. The reasons are as follows: firstly, firms with ESG advantages can obtain government policy preferences and help reduce the innovation cost of firms^51^. Secondly, by taking into account the social value, firms carrying out ESG practice can help build trust in an uncertain environment, meet the needs of employees self-worth realization, attract creative employees to join, form a virtuous circle of internal and external resources, and then promote enterprise innovation^52^. Thirdly, the social responsibility image conveyed by firms helps to improve the risk tolerance of related stakeholders and create a more tolerant environment for enterprise innovation. Fourthly, firms with ESG advantages pay more attention to long-term interests and are willing to abandon short-term profits and increase R&D investment, thereby enhancing their sustainable development capabilities. Therefore, ESG performance will promote the improvement of enterprise value through the efficiency of enterprise green innovation.

Based on this, this study proposes the following hypotheses H1:

#### Hypothesis H1

Given other conditions, the better the ESG performance, the better the stock return performance, and the stronger the enterprise's systemic crisis response capability.

## Empirical design

### Sample and data sources

This study comprehensively uses the event study and the multiple regression method to examine the value effect of ESG performance under the impact of major public health events. The COVID-19 provides a valuable opportunity window for this study to examine the crisis response ability of firms.

In terms of sample selection, this study takes January 20, 2020 as the event day, and takes the companies listed on A-shares before January 20 as the sample selection range to examine the impact of ESG performance on the short-term stock market performance of firms. On January 20, 2020, Academician Nanshan Zhong announced that there was a human-to-human phenomenon of COVID-19. With the beginning of the "Seal City" campaign in Wuhan, other cities followed suit, and the national people's attention to the novel coronavirus epidemic and the resulting negative emotions reached the highest point. The day was also the highest stock yield during the epidemic, and then began to plummet.

In terms of sample interval selection, this study selected three days before and after the outbreak of the health event crisis as the event window, selected 260 trading days before the event date to the first 30 trading days [− 260, − 30] as the estimation window, and selected other window periods (including 10 days before and after the event date), other event occurrence days (such as February 3, 2020, which is the first day of opening after the end of the Chinese Spring Festival) for robustness test.

The ESG performance data in this study mainly come from the CSMAR database and company annual report disclosures. If there are any discrepancies in the data, the company's annual report shall prevail. Other financial data of the company come from CSMAR database. In order to ensure the reliability of the data, this study eliminates the samples as follows: (1) ST and PT companies; (2) Considering the integrity of the data, samples of listed companies with incomplete financial data are eliminated; (3) Refer to Kothari Warner^53^ study, the estimation window is defined as [− 260, − 30], so samples with an estimation window of less than 230 trading days are eliminated to avoid the normal rate of return being interfered by other factors; (4) Considering that the performance indicators of financial companies are not comparable to those of general companies, financial companies are excluded. Finally, 2993 valid cross-sectional samples were obtained. The data on ESG performance and control variables are based on the 2019 annual report data disclosed by the company. The main reason is that the public emergency will not affect the company's financial reporting in 2019, so the company data in 2019 is a relatively clean research sample. To avoid the influence of outliers, continuous variables are winched by 1%.

### Variable definition

#### Measuring the systemic crisis response capability of firms

The "response" in crisis response capabilities includes two aspects, namely preventing crises that will occur and dealing with crises that have already occurred. "Capability" refers to an enterprise’s ability to resist, recover, and adapt in the face of crises. This is an important reason why different firms have different performance in facing crises^21^. Therefore, an enterprise's systemic crisis response capability is an enterprise's ability to respond to external disturbances, resist shocks or disturbances, and adjust its own development path. It is a kind of enterprise resilience or enterprise value.

The measurement of corporate resilience mainly uses corporate financial indicators (such as operating income growth rate, total asset growth rate, investment efficiency, etc.) or market indicators (such as stock price decline, stock price volatility, etc.)^31,54^. The sample period of this study is relatively short, and financial indicators are difficult to reflect in a short period of time, so it is more appropriate to use corporate market indicators in this study. This study draws on the practices of Pan and Xu^55^, Albuquerque et al.^31^ and Shan et al.^3^ uses the cumulative excess return (CAR) calculated by the market adjustment model method to measure the enterprise's systemic crisis response capabilities. The smaller the CAR value, the more serious the loss of corporate value under the impact of the crisis. The larger the CAR value, the more moderate the loss of corporate value under the impact of the crisis, indicating that the enterprise's ability to respond to systemic crises is stronger in the short term.

**Figure 1:**
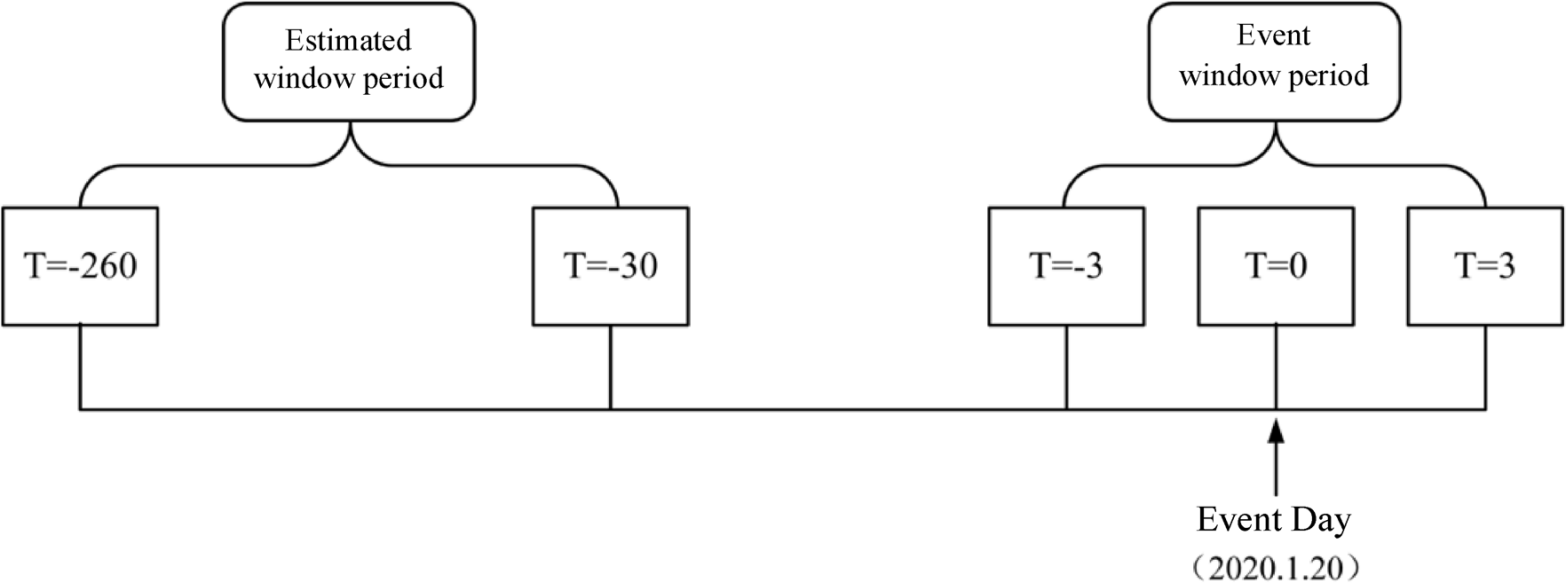
The event research method defines the window period and the event day diagram.

First, this study needs to determine the estimation period and window period. Following the approach of Xiao et al.,^25^, January 20, 2020 is used as the event day and is recorded as *t* = 0. That day was the highest point for stock returns during the epidemic, and then began to plummet. Because the epidemic responds quickly as an emergency, the event window date is selected as [− 3, 3], that is, 3 trading days before and after the event date, for a total of 7 trading days. In order to obtain an estimation window of reasonable length, referring to the research of Kothari and Warner^53^, the estimation window is defined as [− 260, − 30] to avoid the normal return rate being interfered by other factors. The diagram is shown in Fig. 1.

Secondly, this study uses the following market model to calculate the expected return rate of individual stocks:

1 $${r}_{it}={\alpha }_{i}+{\beta }_{i}{r}_{Mt}+{\varepsilon }_{it}$$

In Eq. (1), *r*_*it*_ is the rate of return of stock i on the t-th trading day, *r*_*Mt*_is the A-share market comprehensive return index on day t, ${\varepsilon }_{it}$ is the random error term, ${\alpha }_{i}$ and ${\beta }_{i}$ is the parameter to be estimated.

After estimating the risk-free rate of return $\widehat{\alpha }$ and $\widehat{{\beta }_{i}}$ through Eq. (1), the excess rate of return on the t-th trading day can be expressed as:

2 $${AR}_{it}={r}_{it}-{\beta }_{i}{r}_{Mt}-\alpha$$

Calculate the average daily excess return ARR of each company, which can be expressed as:

3 $${ARR}_{it}=\frac{1}{N}\sum_{i=1}^{N}{AR}_{it}$$

Finally, this study calculates the cumulative excess return within the event window period [− t, t], and the calculation formula is:

4 $${CAR}_{i[-t,t]}=\sum_{T=-t}^{t}{AAR}_{it}$$

#### ESG

This study uses Huazheng's ESG rating as the proxy variable for ESG, which has been widely recognized by the industry and academia due to its advantages such as long evaluation backtracking period, wide coverage, and evaluation system that is in line with China's national conditions^56^. The ESG rating indicators of Huazheng include 9 levels, including C, CC, CCC, B, BB, BBB, A, AA, and AAA. In order to facilitate measurement, this study assigns ESG ratings of listed companies from low to high to 1–9, respectively.

#### Control variable

In order to alleviate the estimation bias caused by the omitted variables, this study refers to the research of Albuquerque et al.^31^ and Xiao et al.^25^, and introduces relevant control variables involving company characteristics: size, asset-liability ratio, nature of corporate ownership, board size, Tobin's Q, corporate cash level, etc. The variables and symbols involved in this study are shown in Table 1.

**Table 1:** Main variable definition.

Symbol	Definition
CAR	Accumulated excess return rate within the day of event occurrence. The event occurred on January 20, 2020, with a window period of (− 3, + 3), and an estimated window period of [− 260, − 30]
ESG	Assignment of ESG ratings 1–9
Size	Natural logarithm of total assets
Lev	Total liabilities/total assets
Roa	Net profit/total assets
Soe	State owned enterprise value is 1, otherwise it is 0
Q	Market value/asset replacement cost
Board	The natural logarithm of the number of board members
Cashflow	Ratio of net cash flow generated from current operating activities to total assets at the end of the period
Industry	Industry dummy variables

### Model

This study controls industry fixed effects and uses clustered robust standard errors to control for the impact of unobservable factors. The model settings are as follows:

5 $${CAR}_{i}={\alpha }_{i}+{{\beta }_{i}ESG}_{i}+\sum {\gamma }_{k}{Firm\_control}_{k,i}+\delta {Industry}_{i}+{\varepsilon }_{i}$$

In Eq. (5), the subscript i represents the listed company code; *CAR*_*i*_ represents the market reaction of individual *i*. $ESG$_*i*_ represents the ESG performance, and *Firm_control* represents the company’s control variables. The data on ESG performance and control variables in this study are measured using 2019 data. This treatment has several advantages. First, it is consistent with the theme of this study. Because this study attempts to examine the role that the company's ESG performance and other characteristics before the crisis play in the company's ability to withstand the crisis when unexpected events cause the company to fall into crisis, that is, the value effect on stabilizing the company's stock price. Second, it can avoid endogeneity problems. After the crisis, many companies will temporarily perform corporate social responsibilities and issue ESG reports. In order to avoid the endogenous problems caused by this situation, this study only considers the mitigating effect of company characteristics before the epidemic on sudden shocks. Since there are differences in the severity of crisis shocks in different industries^57^, this study controls industry fixed effects.

## Baseline and robust results

### Descriptive statistics

The descriptive statistics of this study show that the mean and median CAR are − 0.1% and − 1% respectively (see Table 2), which means that the occurrence of the COVID-19 has sent a negative signal, causing a "confidence gap" for investors, making investors pessimistic about the future stock market, thus causing a negative impact on stock prices. The maximum value loss reached 12.8%, while the maximum value increase could reach 22.8%, indicating a significant gap in the cumulative excess return rate of firms during the epidemic period. The minimum value of ESG performance is 0 and the maximum value is 8. The ESG performance of different companies varies greatly. The logarithmic mean of firm size is 21.010. The average level of corporate asset-liability ratio is 31.3%, and there is a large gap between the minimum and maximum values, indicating that different companies have different asset-liability levels. State-owned firms accounted for 32.5%. The minimum value of corporate cash flow holding levels is − 22.4%, and the maximum value is 26.6%, indicating that there are certain differences in cash flow levels among sample companies. The average and median levels of corporate cash flow holdings are 3.9% and 4.2% respectively, indicating that listed companies cash holding levels are generally low. The mean value of profitability is 6.4%. It can be seen from the descriptive statistics of the variables that the values of each variable are within a reasonable range, which is consistent with previous research, and there are no extreme values.

**Table 2:** Descriptive statistics.

Var name	Obs	Mean	SD	Min	Median	Max
CAR	2993	− 0.001	0.056	− 0.128	− 0.010	0.228
ESG	2993	4.115	1.298	1.000	4.000	8.000
Size	2993	21.010	0.915	19.236	20.839	25.966
Lev	2993	0.313	0.176	0.027	0.295	0.905
ROA	2993	0.064	0.188	− 9.117	0.072	4.489
Board	2993	2.232	0.179	1.609	2.303	2.944
Soe	2993	0.325	0.469	0.000	0.000	1.000
Q	2993	1.427	1.511	0.059	1.084	32.246
Cashflow	2993	0.039	0.075	− 0.224	0.042	0.266

### Mean differences inspection

According to the median of ESG performance, the sample is divided into high ESG performance group (greater than the median) and low ESG performance group (not greater than the median). According to the mean difference test results in Table 3, it can be seen that the cumulative excess return rate of the high ESG performance group during the window period is 0.3%, which is higher than the mean of the low ESG performance group − 0.5%, and there is a significant difference at the 1% level. This indicates that during the crisis, the market responded more positively to companies in the high ESG performance group, preliminarily supporting hypothesis H1a that ESG performance has the ability to withstand crises during the crisis. Groups with poor ESG performance will have more serious negative market reactions and greater reductions in corporate value. In addition, groups with good ESG performance have higher ROA, smaller board size, lower proportion of state-owned firms, and higher corporate cash holdings.

**Table 3:** Mean differences inspection.

Var name	Groups with poor ESG performance	Mean	Groups with good ESG performance	Mean	Mean diff	t
CAR	1219	− 0.005	1774	0.003	− 0.008***	− 3.887
Size	1219	21.027	1774	20.998	0.029	0.844
Lev	1219	0.343	1774	0.292	0.051***	7.910
Roa	1219	0.069	1774	0.087	− 0.018***	− 10.601
Board	1219	2.241	1774	2.225	0.016**	2.424
Soe	1219	0.469	1774	0.227	0.243***	14.389
Q	1219	1.284	1774	1.525	− 0.241***	− 4.304
Cashflow	1219	0.035	1774	0.046	− 0.012***	− 0.012***

“*”, “**” and “***” indicate statistical significance at the 10%, 5% and 1% levels, respectively.

### Correlation analysis

Table 4 shows the results of Pearson correlation analysis between the main variables. The CAR value and ESG performance are significantly positively correlated at the 1% level, providing preliminary evidence that ESG performance affects corporate value. The lower the enterprise's asset-liability ratio, the stronger its profitability, the smaller the board of directors, and it is a non-state-owned enterprise, the higher the enterprise's stock return rate under the impact of the crisis. Since the correlation coefficients between each variable are less than 0.5, and except for enterprise size, other control variables used are significantly correlated with CAR at the 5% level. It is initially verified that the control variables introduced are effective and not there is severe multicollinearity.

**Table 4:** Correlation analysis.

	CAR	ESG	Size	Lev	ROA	Board	Soe	Q	Cashflow
CAR	1								
ESG	0.043**	1							
Size	− 0.033*	0.185***	1						
Lev	− 0.088***	0.0160	0.332***	1					
ROA	0.105***	0.077***	− 0.035*	− 0.544***	1				
Board	− 0.052***	0.037**	0.163***	0.134***	− 0.097***	1			
Soe	− 0.085***	0.134***	0.207***	0.291***	− 0.280***	0.245***	1		
Q	0.071***	− 0.068***	− 0.211***	− 0.235***	0.230***	− 0.138***	− 0.210***	1	
Cashflow	0.0170	0.105***	0.099***	− 0.131***	0.321***	0.058***	0.082***	0.107***	1

“*”, “**” and “***” indicate statistical significance at the 10%, 5% and 1% levels, respectively.

### Benchmark regression

Table 5 reports the results of the benchmark regression of ESG performance on excess stock returns. Among them, columns (1) and (2) use ESG performance as the independent variable. Column (1) controls no other variables except industry fixed effects. From regression (1), we can see that companies with high ESG performance have better market reactions in stock prices during crises. When ESG performance increases by one standard deviation (1.298), the company's stock price return increases by 23.36% (0.180*1.298 = 0.2336), indicating that ESG performance can indeed reduce the firm's value loss caused by the impact of the crisis. In column (2), this study controls for a series of firm-level influencing factors. The coefficient of ESG is 0.186 and is statistically significant at the 5% level. The economic implication of this result is that ESG performance changes by one standard deviation (1.298), and the CAR value of stock excess returns increases by 24.14% on average (0.186*1.298 = 0.2414), indicating that ESG performance can effectively alleviate the company's value damage during crisis shocks.

**Table 5:** Regression estimation of ESG performance and market reaction of stock price.

	(1)	(2)
	CAR	CAR
ESG	0.180** (2.363)	0.186** (2.355)
Size		− 0.021 (− 0.184)
Lev		− 0.796 (− 1.015)
ROA		6.647** (2.334)
Board		− 0.833 (− 1.429)
Soe		− 0.236 (− 1.010)
Q		0.155* (1.809)
Cashflow		− 0.195 (− 0.126)
Constant	1.143 (0.751)	2.568 (0.909)
N	2993	2993
R2	0.038	0.048
Industry	Yes	Yes

Reported in parentheses are t-statistics. “*”, “**” and “***” indicate statistical significance at the 10%, 5% and 1% levels, respectively.

The above results provide evidence that ESG performance improves excess stock returns and verifies the research hypothesis H1 that ESG performance plays an effective role in companies resisting adverse external risks.

### Robustness test

This study uses 5 robustness testing methods to further verify the reliability of the research conclusions in this study. These include introducing the shock effect of new cases, resetting the crisis period window, changing event days, considering samples with special characteristics, and changing dependent variables.

#### Impact effect of introducing new cases

After the COVID-19 epidemic, the capital market has experienced intense shocks, and this shock has not abated in the short term. The event system theory believes that the intensity characteristics of an event determine the degree of impact of the event^58^. In order to examine the continued impact of the epidemic on the capital market, referring to the research of Ding et al.,^29^, this study uses weekly panel data and introduces the variable of the intensity of the epidemic impact. The epidemic impact intensity variable is measured by the cumulative confirmed cases of the new crown, and the new case data comes from the CSMAR database.

In Table 6, Model 1 introduces the interaction term between ESG performance and weekly new cases (Case). Weekly new cases are measured in logarithmic form. Model 2 introduces the interaction term between ESG performance and the growth rate of new cases (Covid). The growth rate of new cases can measure the continued impact of the epidemic. The measurement of this indicator refers to the research of Ling et al.^59^ and the calculation method is: Covidt = [Ln(1 + ∆Caset) − Ln(1 + ∆Casest − 1)], where ∆Casest represents the number of newly confirmed cases in the country on day t. Therefore, Covidt actually calculates the daily growth rate of domestic cases. The dependent variable is the weekly stock return for each firm. It can be seen from the following regression results that the coefficient of the interaction term ESG*Case and ESG*Covid is significantly positive at the 1% level, indicating that ESG performance has a promoting effect on corporate value under the impact of the epidemic.

**Table 6:** Robustness test: the shock effect of introducing new cases.

	(1)	(2)	(3)	(4)	(5)	(6)	(7)	(8)	(9)	(10)
	Considering the impact of the epidemic		[− 10, 10]	[− 10, 10]	[− 3, 3]	[− 3, 3]	Excluding samples from Hubei Province	Control the distance between listed companies and the source of the epidemic	Excluding the pharmaceutical industry	Standard deviation of weekly stock returns from January to March 2020
	Weelky_Storeturn	Weelky_Storeturn	CAR	CAR	CAR	CAR	CAR	CAR	CAR	Wretwd_sd
ESG*Case	0.005*** (2.551)		0.001** (2.425)	0.001*** (2.672)	0.001*** (3.537)	0.001*** (2.913)	0.170** (2.135)	0.186** (2.349)	0.264*** (3.292)	− 0.002*** (− 4.511)
ESG*Covid19		0.002*** (3.631)								
ESG	− 0.002 (− 0.750)	− 0.000 (− 0.818)								
Case	− 0.014*** (− 7.596)									
Covid19		− 0.027*** (− 13.349)								
LDistance								− 0.108 (− 0.720)		
Control	Yes	Yes	No	Yes	No	Yes	Yes	Yes	Yes	Yes
Industry	Yes	Yes	Yes	Yes	Yes	Yes	Yes	Yes	Yes	Yes
N	14,960	20,941	2993	2993	2993	2993	2911	2911	2733	2992
R2	0.040	0.041	0.043	0.054	0.027	0.036	0.048	0.049	0.047	0.066

Reported in parentheses are t-statistics. “*”, “**” and “***” indicate statistical significance at the 10%, 5% and 1% levels, respectively.

#### Resetting the window in crisis period

This study changes the window period to [− 10, 10] for regression analysis. The results are shown in Table 6. It can be seen from the regression results that the ESG performance coefficient is still significantly positive at the 5% level. When ESG performance increases by one standard deviation, the stock's cumulative abnormal return increases by 0.13% (0.001*1.298). Overall, the results of this study remain robust by changing the event window period and the measurement method of ESG performance. The better the ESG performance, the better the company's market performance during the crisis.

#### Replacement event day

This study changes the event date to February 3, 2020, which is the first day the stock market opens after the Spring Festival. The stock market is a market composed of many investors participating in games, so investor sentiment is considered an important factor affecting the stock market. Considering that it takes a process for positive emotions to arise, generally affect the market, and finally subside, it is impossible for the "general" emotional surge before the Spring Festival to skyrocket in one day and then subside on the same day. Therefore, this study uses the first day after the Spring Festival as the event day, that is, [− 3, 3] as the event window. The research results are shown in Table 6. ESG performance significantly improves corporate systemic crisis response capabilities.

#### Considering the special samples

This study takes into account the particularity of the sample: (1) The particularity of the sample in Hubei Province: Since the epidemic started in Wuhan, it is the area most severely affected by the new coronavirus epidemic, and then gradually spread to surrounding provinces and other provinces. Hubei Province is very different from other regions in terms of the number of confirmed cases, the number of deaths, and the emergency response time. In order to avoid the results being affected by special samples, this study excludes samples from Hubei Province for robustness testing. (2) Since the closer to the origin of the epidemic, the greater the impact on the company, the distance between listed companies and Wuhan, Hubei, the origin of the epidemic, is controlled. (3) The pharmaceutical industry samples are special: the pharmaceutical industry tends to perform better during the epidemic. Therefore, this study conducts regression by excluding samples from the pharmaceutical industry. The results in Table 6 show that after excluding samples from Hubei Province, after controlling the distance from listed companies to Wuhan, the origin of the epidemic, and after excluding samples from the pharmaceutical industry, the regression results are still robust.

#### Replace dependent variable

This study replaces the dependent variable and uses the standard deviation of weekly returns of listed companies from January to March 2020, as shown in Table 6. When stock returns fluctuate greatly, it means that the company's resilience is poor in the short term. ESG is negatively correlated with Wretwd_sd at a significant level of 1%, indicating that ESG performance reduces stock market volatility and corporate resilience is good. ESG performance improves a company's ability to withstand adverse shocks.

## ESG performance and systemic crisis response capabilities: analysis of the mechanism of action

In the previous analysis, through rich identification strategies and robustness analysis, we answered the question of whether the development of ESG performance affects the company's ability to respond to systemic crises (stock returns). But in the face of sudden crisis events, why can ESG performance increase its stock returns? If its mechanism of action can be clarified, it can better provide reference for corporate decision-making.

### Mechanism analysis

#### Financing constraints channels

A company's higher level of cash holdings can provide a buffer for capital flow shortages and avoid higher external financing costs and financial crisis costs due to sudden shocks and adverse fluctuations^60^. Research by Xiao et al.^25^ shows that cash held by firms during the crisis played a preventive role and promoted the cumulative excess rate of return of firms. This study uses the financing constraint index KZ and corporate operating cash flow level as the measurement indicators of the degree of financing constraints for testing. The results in Table 7 show that ESG performance significantly eases the degree of financing constraints and improves the level of cash flow from operating activities. This is actually not difficult to understand. First of all, according to the signaling theory, companies with good ESG performance show positive signals of the company's sustainable development capabilities, reducing the uncertainty faced by investors^47^. Secondly, corporate social responsibility is conducive to obtaining key strategic resources from stakeholders^48^, conveying more corporate-specific information to creditors such as banks, and opening up channels for funding sources, thus alleviating corporate financing constraints. In addition, in the context of China's "carbon peak" and "carbon neutrality", companies with good ESG performance will receive more policy support, thereby easing financing constraints^49^. Liquidity is the key to an enterprise's ability to respond to crises during the epidemic. Crisis response is a resource-consuming activity, and abundant cash is conducive to playing an insurance role. Therefore, ESG performance can improve the company's crisis response capabilities through the financing constraint mitigating effect.

**Table 7:** ESG performance and financing constraints mitigation channels in corporate systemic crisis response.

	(1)	(2)	(3)	(4)	(5)
	KZ_index	Cashflow	lnGreInvia	lnGreUmia	lnGreTotal
ESG	− 0.221*** (− 11.109)	0.004*** (3.695)	0.099*** (4.206)	0.040* (1.888)	0.095*** (3.745)
Controls	Yes	Yes	Yes	Yes	Yes
Industry	Yes	Yes	Yes	Yes	Yes
N	2992	2993	1123	1123	1123
R2	0.195	0.183	0.130	0.118	0.106

Reported in parentheses are t-statistics. “*”, “**” and “***” indicate statistical significance at the 10%, 5% and 1% levels, respectively.

#### Green innovation efficiency channels

O'Reilly and Chatman^61^ believe that creativity and innovation norms may be the most effective mechanisms to promote organizational adaptability in major crises. Innovation, as a strategic measure, is a prerequisite for ensuring a company's survival in times of environmental change and turbulence, and is a powerful source of performance growth^62^. Facing the impact of the crisis, the research and development of new products has a strong resistance to the crisis. When firms are in a turbulent environment, it is of great significance for firms to adapt to the changing environment through research and development to improve their competitiveness.

Therefore, this study refers to the practice of Wang Xin and Wang Ying^63^ and uses green invention patents (lnGreInvia), green utility model patents (lnGreUmia) and the total number of green patents (lnGreTotal) to measure corporate green innovation capabilities. From the regression results in Table 7, we can see that ESG performance significantly improves corporate green innovation capabilities. This is because ESG guides companies to adopt more advanced, safe, energy-saving and environmentally friendly production techniques and processes to develop green and energy-saving products^64^, which will promote the improvement of corporate resource allocation efficiency. Green innovation output can promote firms to develop solid competitive advantages and sustainable competitiveness in order to win forward-looking technologies in an uncertain environment, thereby enhancing corporate value.

### Heterogeneity analysis

Different companies (industries) have been affected by the epidemic to different extents, and there will be significant differences in the improvement of CAR value. This study then examines the role of ESG performance on CAR from four aspects: enterprise size (Size), nature of enterprise property rights (SOE), market competition level (HHI), and CEO power (CEOPW).

#### Heterogeneity analysis of firm size

Based on signaling theory and expectation theory, the public, especially consumers and external investors, believe that the social responsibility fulfillment mechanism of large firms is more standardized, institutionalized and normalized. Due to resource constraints, small and medium-sized firms tend to use limited resources to solve key issues such as survival and development^65^. Stereotypes have been formed about the ESG practices of companies of different sizes, and they have a "natural" favorable impression and expectation of large companies. Therefore, this study examines whether corporate size plays a "solidifying" effect on the impact of ESG performance on corporate resilience.

From the perspective of enterprise size, this study multiplies the Size and ESG to test the difference in the impact of ESG performance on CAR under different enterprise sizes. The regression results are shown in column (1) of Table 8. The coefficient of the interaction Size*ESG is significantly negative at the 1% level, indicating that relatively small companies can improve their ESG performance and are more conducive to improving corporate crisis response ability. The good ESG performance of small-scale companies releases positive signals, increases consumer attention and investor confidence, and reverses the public's perception of mediocre social governance of small companies.

**Table 8:** Heterogeneity analysis.

	(1)	(2)	(3)	(4)
	CAR	CAR	CAR	CAR
ESG	0.027** (2.548)	0.003*** (2.725)	0.012*** (3.533)	0.004*** (3.392)
ESG*Size	− 0.001** (− 2.452)			
ESG*Soe		− 0.003* (− 1.856)		
ESG*Hhi			− 0.076*** (− 3.159)	
ESG*CEOPW				− 0.003** (− 2.290)
Size	0.008*** (3.361)			
Soe		0.010 (1.453)		
Hhi			0.159 (1.102)	
CEOPW				0.018*** (2.751)
Controls	Yes	Yes	Yes	Yes
Industry	Yes	Yes	Yes	Yes
N	2993	2993	2993	2993
R2	0.052	0.049	0.052	0.051

Reported in parentheses are t-statistics. “*”, “**” and “***” indicate statistical significance at the 10%, 5% and 1% levels, respectively.

#### Heterogeneity analysis of the nature of property rights

From the perspective of enterprise ownership, the management of state-owned firms need to take into account political goals while pursuing economic goals, which has caused policy burdens on state-owned firms^66^, and the distraction of business energy has increased the business risks of the firms. Therefore, due to differences in the ownership forms and development goals of firms, as well as the disparity in market competitiveness of firms with different property rights, the nature of property rights may serve as a heterogeneous factor that affects the relationship between ESG levels and corporate crisis response capabilities.

The regression results are shown in column (2) of Table 8. Compared with state-owned firms, ESG performance improves the value of non-state-owned firms more obviously. The possible reason is that state-owned firms themselves have advantages such as talent reserves, financing costs and policy preferences. Non-state-owned firms have stronger motivation and willingness to engage in ESG activities and disclose them in order to gain favor from the market. Good ESG performance can help meet or even exceed investor expectations. Therefore, ESG advantages have a smaller impact on the value of state-owned firms, but will have a greater impact on the value of non-state-owned firms.

#### Heterogeneity analysis of market competition degree

When industry competition becomes fierce, companies' bargaining pressure increases, market share is squeezed out, and profit margins are compressed. Especially during the COVID-19 epidemic, social and economic stagnation has greatly impacted product demand, and the pressure on companies to survive has increased sharply. In order to survive in competition, the information cost savings and rapid strategic adjustments brought about by ESG performance will improve the stability of firms in the product market, upstream and downstream industry chains, and internal organizations^36^, and improve the enterprise's ability to respond to shocks.

From the perspective of market competition level, this study multiplies HHI with ESG to test the difference in the impact of ESG performance on CAR under different levels of market competition. HHI is calculated using the Herfindahl Index calculated from the operating income of companies in the industry. The regression results are shown in column (3) of Table 8. The coefficient of the interaction HHI*ESG is significantly negative at the 1% level, indicating that the promotion effect of ESG performance on CAR is more significant in an environment with fierce market competition.

#### Analysis of the heterogeneity of CEO power

Power is very important for CEOs to maintain control of the firm^67^. Agency theory holds that executives often have strong incentives to seize control rights for personal gain, waste corporate resources, and engage in a series of activities that harm corporate value because of their internal information advantages^68^. ESG performance cannot be quantified in the short term, which in turn affects management bonuses linked to operating performance and weakens CEOs' enthusiasm for ESG investments. This study multiplies $CEOpower$ and $ESG$ to test the difference in the impact of ESG performance on CAR under different CEO power intensities. The results are shown in column (4) of Table 8. The coefficient of the interaction term $CEOpower$*$ESG$ is significantly positive at the 5% level. Therefore, it can be seen that the promoting effect of ESG performance on CAR is more significant in companies with weak CEO power. This may be because the concentration of power among managers can lead to severe opportunistic behavior, and the response to crises becomes less efficient.

### Further analysis

#### The impact of ESG sub items on enterprise resilience

ESG rating is an overall assessment of environment (E), society (S) and governance (G) after taking into account weighted factors. In order to examine the differential impact of the three dimensions of ESG on corporate resilience, this study examines the relationship between each of the three dimensions and corporate resilience. According to the regression results in column (1), (2) and (3) of Table 9, only items S and G, that is, good social performance and governance levels, have a significant positive effect on corporate resilience. And the promoting effect of item S is more significant. A possible explanation for this is that, on the one hand, social responsibility to protect employee welfare, health and other rights and interests is the factor most directly related to employees during the epidemic, and is conducive to the maintenance of corporate stability. On the other hand, compared with environmental factors, governance factors are factors that are controllable by an enterprise and closely related to its profits and development. Therefore, companies invest limited resources in the practice of improving governance in the short term, neglecting environmental investment to a certain extent. Social responsibility factors play the greatest role in improving corporate value during a crisis.

**Table 9:** Further analysis.

	(1)	(2)	(3)	(4)	(5)	(6)	(7)	(8)
	CAR	CAR	CAR	saleempee	lntfp	ROS	CAR	CAR
e_score	− 0.006 (− 0.528)							
s_score		0.026*** (2.606)						
g_score			0.022* (1.774)					
ESG				0.094*** (4.408)	0.033*** (2.665)	0.032*** (4.900)	0.233** (2.183)	0.100 (0.839)
Controls	Yes	Yes	Yes	Yes	Yes	Yes	Yes	Yes
Industry	Yes	Yes	Yes	Yes	Yes	Yes	Yes	Yes
N	2993	2993	2993	1779	1949	2009	1723	1270
R2	0.047	0.049	0.048	0.152	0.531	0.093	0.042	0.068

Reported in parentheses are t-statistics. “*”, “**” and “***” indicate statistical significance at the 10%, 5% and 1% levels, respectively.

#### Analysis of the economic consequences of ESG performance

Under the influence of the crisis, the previous study conducted a series of empirical tests by taking advantage of the forward-looking characteristics of stock returns in the capital market and the rapid response to market information. Since accounting performance indicators are slow to incorporate information, this study conducts further analysis on accounting performance indicators during the crisis.

This study analyzes the economic consequences of ESG performance from the perspective of per capita income generation (saleempee), labor productivity (lntfp) and return on sales (ROS). Per capita revenue generation is measured using operating income per employee. This study uses total factor productivity as a proxy variable for labor productivity. This index rate can reflect the contribution rate to growth of various factors such as enterprise system optimization, management level and technology introduction in addition to the number of enterprise factor inputs. From the column (4), (5) and (6) in Table 9, ESG performance significantly increases per capita revenue generation, labor productivity and return on sales. The impact of the epidemic has brought opportunities for capital expansion to some high-quality companies, increasing firm value.

#### Distinguish the impact of the epidemic

Considering that China has a vast territory and the impact of the epidemic on different regions is significantly different, this study further examines whether the importance of ESG performance is different under the severity of the impact of the epidemic in different regions. Drawing on the research of Xiao et al.^25^, this study divides regions into two categories: high and low epidemic levels. Registration places in Hubei, Guangdong, Henan, Zhejiang, Hunan, Anhui, Jiangxi, Shandong and Jiangsu provinces are divided into groups with higher epidemic impact. If the registration place is in other provinces, they will be divided into a group with a lower impact of the epidemic. The Column (7) and (8) of Table 9 represent the group with severe epidemic and the group with mild epidemic, respectively. The results show that in areas that are more severely affected by the epidemic, the impact of ESG performance on corporate resilience is more prominent, while for companies in the group that are less affected by the epidemic, the impact is smaller and not significant. The above results show that when companies are more severely affected by the epidemic, they are more able to reflect the role of ESG.

## Conclusion and suggestion

The COVID-19 epidemic has had a major impact on society and the economy, causing companies to go through a difficult journey. A large number of companies have fallen into crisis due to the impact of the epidemic. But this crisis also provides an opportunity to test how companies can strengthen their ability to withstand adverse shocks. This study uses the natural experiment provided by the COVID-19 crisis that broke out in 2020 to study the market reaction of the ESG performance of Chinese listed companies to stock prices. Through rigorous empirical analysis, the following conclusions are drawn:

There is a significant positive causal relationship between ESG performance and the cumulative excess return within the event window period. ESG performance affects the company's ability to respond to systemic crises by easing corporate financing constraints and improving corporate risk tolerance. Heterogeneity analysis shows that ESG performance is stronger in small-scale firms, state-owned firms, and in fiercely competitive market environments, with stronger ability to respond to systemic crises. A powerful CEO will weaken the ability of ESG performance to respond to corporate systemic crises. Further research found that only items S and G, that is, good governance level and social performance, have a significant positive promoting effect on corporate resilience. This study analyzes the economic consequences of ESG performance and shows that ESG performance can significantly increase per capita income generation, labor productivity, and sales profit margins. This study also verifies that in areas more severely affected by the epidemic, ESG performance may be more important.

This study puts forward the following policy recommendations: first, for non-state-owned firms and small-scale firms, ESG is a precise and effective policy means to establish a sustainable development concept and accelerate green transformation, and can significantly enhance the market competitiveness of firms. Firms should accelerate the cultivation of ESG concepts, improve the quality and frequency of ESG disclosure, promote ESG governance, integrate the concept of sustainable development into their own development and construction, and create a fair, convenient, competitive and orderly market environment. Second, companies should not over-concentrate the power of management, but should reasonably design the power boundaries of management to encourage them to play an active leadership role in corporate ESG practices. Third, at the policy level, China should speed up the improvement of the scientific nature and reliability of ESG information disclosure standards. Policymakers have strengthened penalties for false ESG construction and combined government guidance measures with corporate voluntary actions to better leverage economies of scale and reduce transformation costs.

## Data Availability

The data used in this article is available upon request. Please contact the corresponding author for access to the data.
